# A Performance Evaluation of Two Hyperspectral Imaging Systems for the Prediction of Strawberries’ Pomological Traits

**DOI:** 10.3390/s24010174

**Published:** 2023-12-28

**Authors:** Tiziana Amoriello, Roberto Ciorba, Gaia Ruggiero, Monica Amoriello, Roberto Ciccoritti

**Affiliations:** 1 CREA—Research Centre for Food and Nutrition, Via Ardeatina 546, 00178 Rome, Italy; 2 CREA—Research Centre for Olive, Fruit and Citrus Crops, Via di Fioranello 52, 00134 Rome, Italy; roberto.ciorba@crea.gov.it (R.C.); ruggierogaia98@gmail.com (G.R.); 3 CREA—Central Administration, Via Archimede 59, 00197 Rome, Italy; monica.amoriello@crea.gov.it

**Keywords:** quality attributes, visible–near infrared system, short-wave infrared system, artificial neural networks, data fusion

## Abstract

Pomological traits are the major factors determining the quality and price of fresh fruits. This research was aimed to investigate the feasibility of using two hyperspectral imaging (HSI) systems in the wavelength regions comprising visible to near infrared (VisNIR) (400−1000 nm) and short-wave infrared (SWIR) (935−1720 nm) for predicting four strawberry quality attributes (firmness—FF, total soluble solid content—TSS, titratable acidity—TA, and dry matter—DM). Prediction models were developed based on artificial neural networks (ANN). The entire strawberry VisNIR reflectance spectra resulted in accurate predictions of TSS (R^2^ = 0.959), DM (R^2^ = 0.947), and TA (R^2^ = 0.877), whereas good prediction was observed for FF (R^2^ = 0.808). As for models from the SWIR system, good correlations were found between each of the physicochemical indices and the spectral information (R^2^ = 0.924 for DM; R^2^ = 0.898 for TSS; R^2^ = 0.953 for TA; R^2^ = 0.820 for FF). Finally, data fusion demonstrated a higher ability to predict fruit internal quality (R^2^ = 0.942 for DM; R^2^ = 0. 981 for TSS; R^2^ = 0.976 for TA; R^2^ = 0.951 for FF). The results confirmed the potential of these two HSI systems as a rapid and nondestructive tool for evaluating fruit quality and enhancing the product’s marketability.

## 1. Introduction

Nowadays, agrifood industries have increased expectations about sustainable production, the delivery of high-quality and safe food products, and the reduction in food losses along the entire supply chain, including post-harvest losses. The quick and accurate monitoring and evaluation of the quality, safety, nutrition, sensorial attributes, and shelf life of food products are being increasingly requested by the agrifood industries. Unfortunately, many industries rely on traditional human visual inspection to monitor product quality. However, this method is inefficient and subjective [1]. Furthermore, traditional and analytical techniques, such as proximate analysis, as well as chromatographic and mass spectrometry, are destructive, expensive, time-consuming, and not useful for large-scale sample evaluation, and they can generate pollution and harmful waste [2,3]. In fact, unlike traditional analytical methodologies for fruit internal quality assessment, the advantages of using hyperspectral imaging systems include rapid analysis times; small sample size; simple sample preparation, usually requiring few steps; reduced costs (compared to other laboratory equipment); and the absence of toxic or carcinogenic chemicals.

In recent years, some smart methods and real-time and nondestructive systems to assess quality parameters have been developed. Among these, computer vision technology and optical techniques have received increased attention for quality assessment. The first technique, based on image processing and analysis, allows for automated visual inspection. However, it can be inefficient in the evaluation of objects with similar colors or for detecting invisible defects. Moreover, it cannot be useful for predicting food chemical composition [1]. On the contrary, vibrational spectroscopy methods, particularly near-infrared spectroscopy (NIRS), have the potential for simultaneously measuring multiple quality properties [1]. However, they are limited in the spatial dimension, without information on position/location of the constituents investigated and could miss contaminants confined to small areas on the products [4].

The combination of spectroscopic techniques and imaging processing gives rise to the hyperspectral imaging (HSI) system, which enables the immediate recognition of different elements and their spatial distribution in the samples in a nondestructive, highly accurate, and fast way with minimal sample preparation. HSI produces a two-dimensional spatial array of vectors, which corresponds to the spectrum at each pixel location. Therefore, a three-dimensional dataset with the two spatial dimensions and one spectral dimension (i.e., a hypercube) is obtained. The spectrum related to each pixel in a food image can represent a fingerprint to identify the biochemical composition of the pixel, thus allowing for the vision of the constituents of the food sample at a pixel level with high accuracy. In fact, HSI can provide simultaneously spatial and physical attributes such as shape, texture, and color of the samples, as well as intrinsic and molecular information [5]. 

HSI has been successfully used to assess fruit quality, fruit ripeness, and fruit damage [6,7,8,9,10,11,12,13,14]. In particular, the rapid and nondestructive evaluation of quality attributes, especially for easily perishable fruit, is a crucial point along all the supply chain. Strawberry fruit (*Fragaria × ananassa* Duch.) is the most consumed berry fruit crop worldwide [15]. It can be consumed fresh or frozen, or utilized for garnishing cakes and pastries or as raw material and additive for jams, juices, ice cream, and jellies [16,17]. However, it is a strongly focused fruit due to its high perishability. Therefore, identifying the qualitative characteristics of the fruit—which are also related to genetic and environmental conditions—to establish the most appropriate time for harvesting, retail sale, and consumption, is of fundamental importance. 

In recent years, machine learning has been successfully applied in precision agriculture and the food industry, especially for the prediction and classification of quality parameters of fruit and vegetables [18]. Among the various machine learning techniques, artificial neural networks (ANN) have a powerful learning ability and identifying and modeling ability for the complex and often nonlinear relationships between input and output signals depending on the provided patterns [19]. Furthermore, ANN can learn from examples datasets through iteration without requiring prior knowledge of the relationships between the process variables. Previous studies proved that ANN can reliably predict fruit characteristics [20,21,22,23].

In light of these considerations, our study aimed to assess the performances in the prediction of two hyperspectral imaging systems with different wavelength ranges, a visible–near-infrared (VisNIR, 400–1000 nm) system and a short-wave infrared (SWIR, 935–1720 nm) system. Prediction models were developed for strawberry firmness, total soluble solid content, titratable acidity, and dry matter using artificial neural network algorithms, highlighting the most predictive input spectral regions for the two systems. Moreover, the fusion of spectral information from visible-light and short-wave infrared bands was used to improve predictiveness of all models. Although previous studies investigated the application of hyperspectral imaging for predicting major strawberry pomological traits [6,14,24], no studies were found to predict dry matter. In general, measurements of several parameters for a single small fruit and berry are quite difficult to carry out, because there is not enough material for a lot of physical and chemical determinations. For this reason, the studies on strawberry often do not consider dry matter coupled with total soluble solid content, titratable acidity, or other phytochemicals. All the pomological traits considered in our study can determine the quality, the price of fresh fruit, and the fruit’s specific intended use. In particular, total soluble solids and titratable acidity can be useful for identifying fruit quality for ready-to-eat purposes, whereas a high content of dry matter is a good indicator for the industrial processing of strawberries into jams, jellies, and juices, among others [25]. This study can provide a valuable contribution to agricultural technology and the food industry by applying hyperspectral imaging for a rapid and nondestructive assessment of strawberry internal quality. Moreover, the identification of the most predictive input spectral regions can be the starting point for building new and inexpensive sensors based on a small number of wavelengths, to be used commercially or for broader practical application.

## 2. Materials and Methods

### 2.1. Plant Material and Experimental Design

Strawberry fruits (*Fragaria × ananassa* L.) from four everbearing strawberry cultivars, listed in Table 1, were bought from local markets in two different periods (April and July).

**Figure 1:**
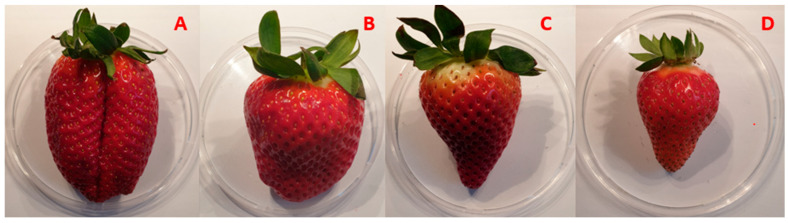
Samples of (**A**) Sabrina; (**B**) Calinda; (**C**) Marimbella; (**D**) Sabrosa.

Samples for each cultivar are shown in Figure 1.

After purchase, the fruits were immediately transported to the laboratory and screened for uniformity, appearance, and the absence of physical defects or decay. Two independent sets of 200 fruit samples, referring to the types of spectroscopic acquisition (VisNIR and SWIR) were constituted. Fifty strawberries were considered for each of the four cultivars (Calinda, Marimbella, Sabrina, Sabrosa).

### 2.2. Analytical Methods

Quality analyses (i.e., weight, length, width, thickness, color (CIELab coordinates: L*, a*, b*), firmness (FF), dry matter (DM), titratable acidity (TA), and total soluble solid content (TSS)) were performed on single fresh fruits, as previously reported by Amoriello et al. (2022) [15]. In detail, digital calliper (±0.05 mm accuracy) was used to determine the strawberry dimensions (length, width, thickness) expressed in mm. Dry matter was evaluated drying the fresh samples at 105 ± 1 °C until a constant weight was reached using oven, and the results were expressed as g 100 g^−1^ of fresh weight (FW). Berry firmness was measured with a penetrometer (Fruit Pressure Tester FT011, TR snc, Forlì, Italy), using an 8 mm tip, and the result was expressed in Newton (N). Skin color was acquired on the external opposite sides of fruit using CIELab color space, obtained with a tristimulus colorimeter (Chroma Meter CR-200; Minolta, Milan, Italy), equipped with a D65 illuminant [15]. 

The method for titratable acidity was based on titration of the acids present in the juice with sodium hydroxide (0.1 N) using an automatic titration system (785 DPM Titrino, Metrohm Ltd., Herisau, Switzerland). Data were given as mEq L^−1^. TSS evaluation was made with a digital refractometer (Refractometer 30PX, Mettler Toledo, Greifensee, Switzerland). TSS was expressed as g 100 g^−1^ of FW.

### 2.3. Hyperspectral Image Acquisition

Two hyperspectral imaging systems (VisNIR and SWIR) were used to acquire hyperspectral images of strawberry samples. 

Images from the first set of samples (VisNIR) were acquired in reflectance mode using a portable Specim IQ hyperspectral camera with push-broom technology (Specim, Spectral Imaging Ltd., Oulu, Finland), as described by Dashti et al. [5]. The VisNIR system included a spectrometer, a CMOS surface detector, a lens (Specimens FX10, Specim, Spectral Imaging Ltd., Oulu, Finland) with a detection range of 400–1000 nm and 224 wavelength points, and a computer system with an imaging acquisition software (Lumo-Scanner, Specim, Spectral Imaging Ltd., Oulu, Finland).

**Figure 2:**
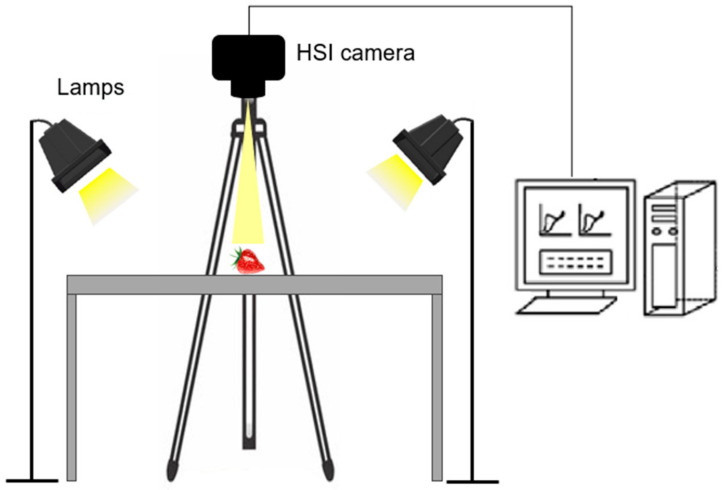
Schematic diagram of the VisNIR hyperspectral imaging system.

The spatial resolution was 512 × 512 pixels and the spectral resolution was 7 nm resulting in 204 spectral bands across the wavelength range. Therefore, a 3D hypercube was obtained with dimensions of 512 × 512 × 204 with over 53 million data points for each scan and active pixel pitch 17.58 µm. The camera was fixed on the tripod facing downwards, and samples were positioned on a table below the camera for scanning (Figure 2). Two halogen-based lamps, placed at 45° facing downwards, were used as the light source. The integration time of the hyperspectral camera was set to 45.0 ms. A white diffuse reflectance target was used to carry out a white reference image to operate in simultaneous modality. The RGB and hyperspectral images were acquired through IQ studio software (Specim, Spectral Imaging Ltd., Oulu, Finland).

Images from the second set of samples (SWIR) were acquired in reflectance mode using a SisuCHEMA Hyperspectral Chemical Imaging Analyser (SPECIM, Spectral Imaging LTD, Oulu, Finland) system. The system uses a push-broom imaging technology that captures one line of an image at a time while scanning the sample on a sliding table. The system included (1) a scanner table having a maximum scanning rate of 60 mm/s and a spatial resolution of 600 μm, with an integrated SPECIM diffusive line illumination unit (located on top of the samples in a 45° angular position with respect to the samples) to illuminate the camera’s field of view; (2) a monochrome InGaAs image sensor detector (Specim FX17, Spectral Imaging Ltd., Oulu, Finland) with a spectral range of 935–1720 nm and spatial resolution of 640 pixels, 224 wavebands and spectral resolution of 8 nm full width half maximum; (3) a computer with an imaging acquisition software (Lumo-Scanner, Specim, Spectral Imaging Ltd., Oulu, Finland). The exposure time of the hyperspectral camera was set to 4.70 ms, the frame rate to 15.20 Hz, the positioning speed of the platform to 20.00 mm s^−1^, and the scanning speed to 5.84 mm s^−1^.

All acquired raw hyperspectral images were then corrected into reflectance hyperspectral images using the white and dark references [26], according to the following equation:

(1) $$I=\frac{I_{raw}-I_{B}}{I_{W}-I_{B}}$$

where I = the corrected reflectance, I_raw_ = the original reflectance, I_B_ = the black reference, and I_W_ = the white reference.

### 2.4. Data Analysis

Two hundred samples for each of the two sets of strawberries were acquired twice, on opposite sides of the fruit. The mean spectrum from two spectra was determined for each sample, baseline corrected and used as a spectral signature of the sample in the spectral dataset. First-order Savitzky–Golay derivative was applied on the data for baseline correction and smoothing. The Savitzky–Golay filter was chosen because it tends to preserve the original signals by removing noise only to some degree [27]. Image segmentation was carried out with the principal component analysis (PCA) algorithm on mean centered spectra, considering the first two principal components (PCs) to remove the background and extract the pixels of each strawberry sample from the entire hyperspectral image. PCA allowed a lower dimensional representation of the data by forming linear combinations of the original wavebands in direction of maximal variance [28]. This is represented by score images. Then, all the hypercubes were transformed into two-dimensional matrices containing as many rows as the pixels retained after background elimination and as many columns as the number of wavelengths by unfolding. The spectra related to all pixels of strawberries were then averaged to obtain the mean spectrum of each sample. This processing was obtained using Evince software (Prediktera AB, Umeå, Sweden).

### 2.5. Prediction Models

Pomological traits of the strawberries (firmness, total soluble solid content, titratable acidity, dry matter) were predicted using artificial neural networks (ANN). A feed-forward architecture of ANN, known as multilayered perceptron (MLP), with back propagation and training algorithms, was employed to build predictive and nonlinear models for the output variables (FF, TSS, TA, DM) for both VisNIR and SWIR systems. Each of two whole datasets, containing the mean spectra of each strawberry sample, was randomly divided into training set (80% of data) and testing set (20% of data). 

**Figure 3:**
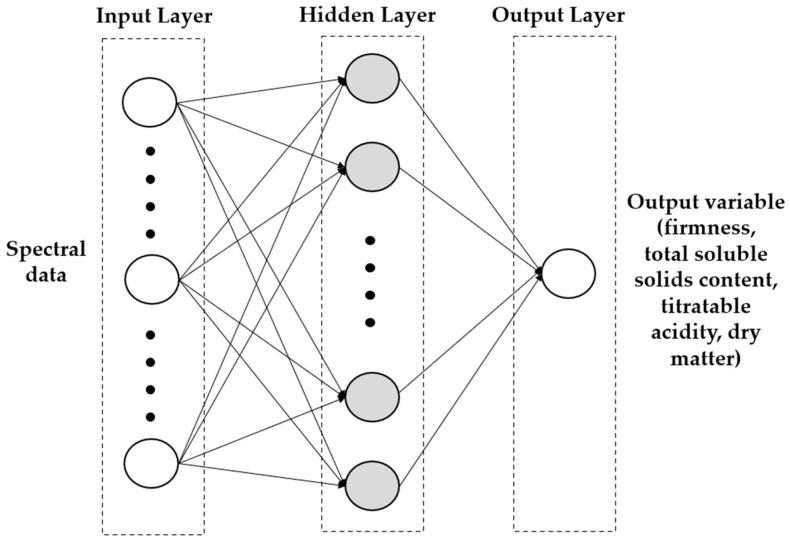
Structure of multilayer perceptron artificial neural network.

The ANN model consisted of three layers: an input layer with the neurons as independent variables (the spectral response), one or more hidden layers, and one output layer for each output variable (FF, TSS, TA, DM) with the neurons as dependent variables, as shown in Figure 3. The number of artificial neurons or nodes equals the size of the input vector. Different activation functions (identity function, logistic function, hyperbolic tangent function, and exponential function), as reported by Amoriello et al. (2022) [15], were considered. Different topologies with different neurons in the hidden layer (from 1 to 25) were tested, and the training process of the network was run 100,000 times with random initial values of weights and biases. The best topology for all pomological traits was evaluated using prediction performance values.

Prediction performances of various ANN configurations for each pomological trait were evaluated using four statistical metrics: the coefficient of determination (R^2^), the mean absolute error (MAE), the root mean squared error (RMSE), and the relative standard error (RSE), as defined by Amoriello et al. (2022) [15].

To improve the performances of models for all quality parameters, the fusion of spectral data from the two HSI systems was applied. It was performed at low-level fusion. Therefore, the two spectral datasets were first autoscaled to compensate for the scale differences, and then concatenated and merged into a single matrix containing 406 variables for modeling. 

Data were processed using TIBCO^®^ Statistica statistical package software (version 13.5, TIBCO software Inc., Palo Alto, CA, USA).

### 2.6. Statistical Analysis

Differences in all measured properties were determined using a one-way analysis of variance (ANOVA) and the Kruskal–Wallis nonparametric test at a significance level of 5%, using SPSS statistical software (version 22, SPSS, Chicago, IL, USA).

## 3. Results and Discussion

### 3.1. Exploratory Analysis

The main quality traits of the first set of strawberry varieties under evaluation are shown in Table 2.

A considerable variability was observed in all pomological traits among the cultivars. Fresh weight ranged from 57 ± 13 g (Sabrina) to 22 ± 10 g (Sabrosa). Consequently, Sabrina and Sabrosa were characterized by the greatest and lowest values of length and width, respectively (Table 2). Data agreed with those reported in several studies on physical characteristics of different strawberry varieties [29,30,31,32]. Fruit color coordinates showed marked differences between Sabrina and Sabrosa. Marimbella and Calinda were between these two cultivars and were very similar to each other from the point of view of color.

On average, no significant differences on firmness were observed among the investigated cultivars. Among genotypes, Sabrosa showed the highest total soluble solid content and titratable acidity (9 ± 2 g 100 g^−1^ FW, and 128 ± 18 mEq 100 g^−1^ FW, respectively; Table 3), whereas the lowest mean values were recorded by Calinda (5.4 ± 0.5 g 100 g^−1^ FW, and 76 ± 12 mEq 100 g^−1^ FW, respectively). Sabrosa also showed the high dry matter content, whereas the lowest was observed in Marimbella (Table 2). These results were in accordance with previous studies reported by several authors [15,33,34].

A similar trend reported above was observed in all investigated attributes also for second set samples, as shown in Table 3.

### 3.2. Image Processing and Spectra Analysis

**Figure 4:**
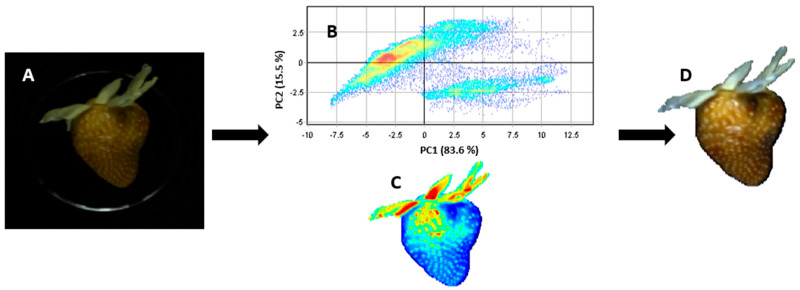
Example of (**A**) strawberry before processing; (**B**) PC1 score plot of pixels; (**C**) PC1 score image; (**D**) RGB image after processing.

Firstly, the unfolding was applied: the hypercube, in which rows and columns represent pixel position in the image and the third dimension of the spectral variables, was reorganized in a bi-dimensional structure for the PCA to be enforced. The new 2D data matrix had pixels as rows and variables as columns. After PCA, was possibly to come back to the original structure by applying the refolding procedure. Figure 4 showed an image of a strawberry before and after the processing phase. The filter application and the removal of pixels corresponding to the background, leaving only the fruit, were shown. A score plot of the first two PCs, which explained more than 98% of variance for all images, was represented. Moreover, a refolded hyperspectral image of strawberry represented by the first principal component score was shown.

**Figure 5:**
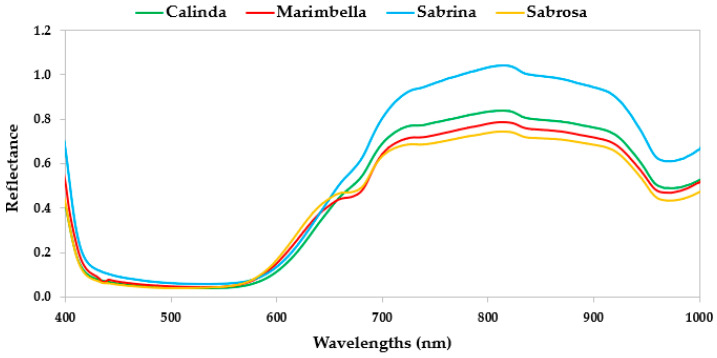
Mean reflectance spectra of strawberries for the four cultivars at wavelength range of 400–1000 nm (VisNIR).

**Figure 6:**
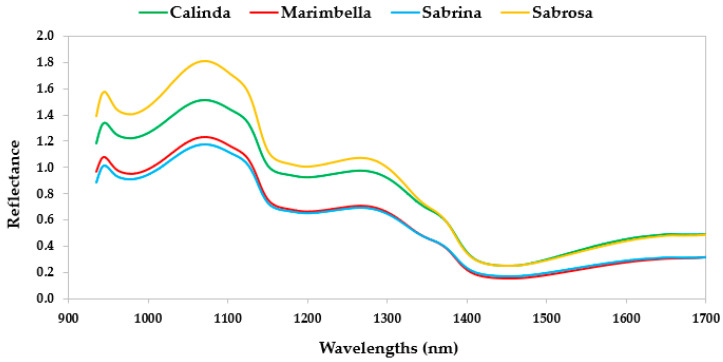
Mean reflectance spectra of strawberries for the four cultivars at wavelength range of 935–1720 nm (SWIR).

The average mean spectra for the four cultivars are shown in Figure 5 and Figure 6. In general, VisNIR spectra (region from 400 to 1000 nm) (Figure 4) is featured by vibration overtones and combination bands of O–H, C–H, and N–H bonds related to the principal structural organic molecules [35]. Many studies on fruit characteristics revealed the presence of the O–H overtone at and 970 nm related to the water [36,37]. In addition, other authors [38,39,40] also reported a strong water absorption peak in fruit samples at around 960 nm. However, Benelli et al. (2022) [35] highlighted a reduced marking and amplitude of the water absorption peaks between 700 and 1000 nm, making the spectral information of the substances at low concentrations in the fruit less covered by the presence of water [35]. Regarding carbohydrates, the absorbance peaks were found in the spectra regions between 950 and 1000 nm, characterized by the O–H and N–H second overtone, the O–H bonds’ combination band, and the C–H third overtone [41]. Pu et al. (2016) [39] indicated a probable sugar absorption band at 840 nm. Fruit pigments can be evaluated based on their spectral characteristics. The wavelength range from 420 to 503 nm is characterized by adsorption of carotenes and xanthophylls [36]. The fruit pigment beta-carotene strongly absorbs near to 475 nm, while the xanthophylls lutein and violaxanthin strongly absorb at approximately 435 nm, with strong absorption across the 350–500 nm range [42]. Sabrina showed a slightly different behavior in this spectral region in comparison with the other cultivars. An anthocyanin pigment sugar–protein complex leads an absorption at around 530–550 nm [43,44]. The spectral reference for chlorophyll absorbance is the peak at 680 nm [42]. Strawberries’ spectra revealed differences at around 680 nm: Marimbella and Sabrosa showed a reduced chlorophyll content in comparison with the other cultivars. This different reflectance data variability among cultivars was likely caused by different levels of fruit ripeness. Moreover, the peak of reflectance for strawberries had occurred at around 800–840 nm, as noted by Tallada et al. (2006) [45].

Regarding the SWIR spectra region, the most prominent reflectance peaks in the regions between 1000–1150 nm are characterized by C–H and O–H functional groups, common in major constituents (e.g., water, sucrose, and cellulose) within the samples [46]. Other vibrational bands for C–H functional groups were also reported in the region from 1300 to 1400 nm. An O–H overlapping with the first overtones of the O–H stretching modes of self-associated and water-bonded O–H functional groups could be found from 1400 to 1600 nm [46]. The local reflectance trough, which appears at approximately 1200 nm, is due to the presence of water in the sample. The reflectance peaks at 1060 and 1270 nm (C–H stretching second overtone) are due to sucrose and cellulose, respectively [46].

### 3.3. Prediction of Pomological Traits

Predictive models for pomological traits were developed using the artificial neural networks. 

Different ANN configurations were tested and compared with each other to determine the optimal MLP architecture (input–hidden–output layers). The network included 204 wavelengths for the VisNIR dataset and 224 wavelengths for the SWIR dataset as input data in the first layer, and one output layer that represented the strawberry parameters. Hidden neurons in the hidden layers were set to a range between 1 and 25. The best configuration for each pomological trait, i.e., the best goodness of fit of ANN models, was highlighted by the lowest RMSE of the training and test sets. 

Table 4 and Table 5 show the neural networks’ architectures according to their topologies, including the MLP algorithm; the numbers of neurons in input, hidden, and output layers; the hidden and output neurons’ activation functions; and the regression metrics (R^2^; RMSE; MAE and RSE) for the highest training and test set predictions for each pomological trait (FF, TSS, TA, DM). The best topology for each attribute was found using neurons in the hidden layer from 1 to 25. The same procedure was applied to both VisNIR and SWIR datasets. The best five ANN architectures for each parameter were shown in Tables S1 and S2 (Supplementary Materials).

Regarding the VisNIR dataset, the best model for berry firmness was carried out with 11 neurons in the hidden layer, a logistic activation function for the hidden neurons, and identity activation function for the output neurons (Table 4). The optimal model for total soluble solid content was developed with 22 neurons in the hidden layer, an exponential activation function for the hidden neurons, and a hyperbolic tangent activation function for the output neurons. The best model of titratable acidity had 16 neurons in the hidden layer, and an identity activation function for the hidden neurons, and an identity activation function for the output neurons. The best model for dry matter was characterized by 19 neurons in the hidden layer, an exponential activation function for hidden neurons, and an exponential function for output neurons.

**Figure 7:**
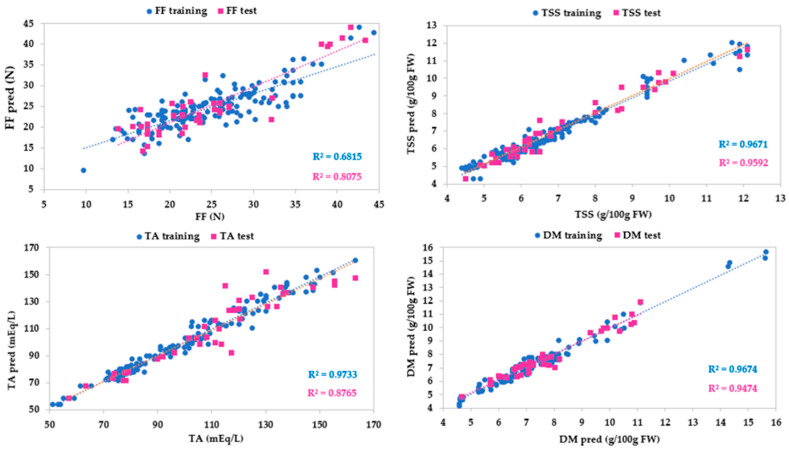
Predicted vs. experimental values of the firmness (FF), total soluble solid content (TSS), titratable acidity (TA), dry matter (DM) using the optimal ANN topologies and VisNIR spectra. The coefficients of determination (R^2^) for training and test sets are reported.

Table 4 and Figure 7 show a high goodness of fit for all parameters, especially for total soluble solid content and dry matter, and with the exception of firmness. In fact, the TSS model had coefficients of determination close to 1 (R^2^ = 0.967 for the training test and R^2^ = 0.959 for the test set) and the other metrics close to 0, indicating a low dispersion of residuals. Likewise, DM also had very high coefficients of determination (R^2^ = 0.967 for the training test and R^2^ = 0.947 for the test set) and very low other metrics. Optimal values of the coefficients of determination of TA (R^2^ = 0.973 for the training test and R^2^ = 0.877 for the test set), MAE, RMSE, and RSE (0.019, 3.922, and 0.019 for the training set, and 1.003, 9.306, and 8.438 for the test set, respectively) showed a high ability in predicting the strawberry titratable acidity. Finally, a poor goodness of fit resulted for firmness, due to low coefficients of determination (R^2^ = 0.682 for the training test and R^2^ = 0.808 for the test set) and high values of MAE, RMSE, and RSE. The low performance of the FF model may be due to two reasons. At first, FF measurements were performed with a manual penetrometer, and therefore were subject to associated operator error. Secondly, the calibrations were carried out using samples at the commercial harvest stage, with FF values between 45 and 10 N. Optimal calibrations for FF should be built by a wider range of variability of FF values, also including samples at different levels of ripeness.

Regarding the SWIR dataset, the best model for firmness was carried out with 10 neurons in the hidden layer, a logistic activation function for the hidden neurons, and an identity activation function for the output neurons (Table 5). The optimal model for total soluble solid content was obtained with 11 neurons in the hidden layer, a logistic activation function for the hidden neurons, and an identity activation function for the output neurons. The best model of titratable acidity had 19 neurons in the hidden layer, and an identity activation function for the hidden neurons, and an identity activation function for the output neurons. Finally, the best model for dry matter was characterized by 19 neurons in the hidden layer, an exponential activation function for hidden neurons, and an exponential activation function for output neurons.

**Figure 8:**
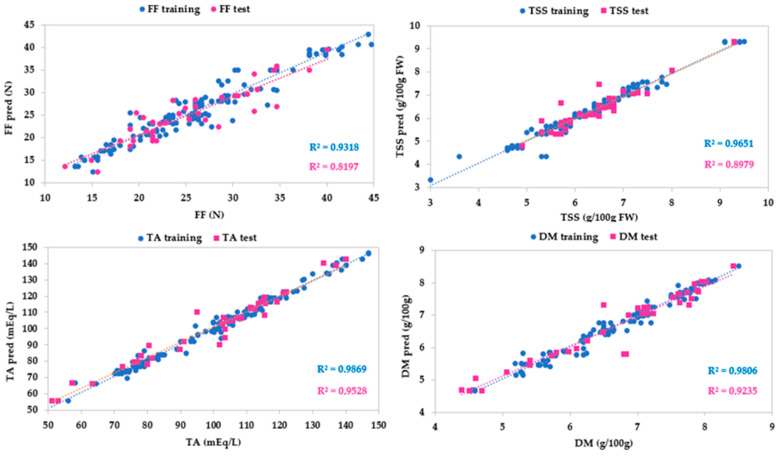
Predicted vs. experimental values of the firmness (FF), total soluble solid content (TSS), titratable acidity (TA), dry matter (DM) using the optimal ANN topologies and SWIR spectra. The coefficients of determination (R^2^) for training and test sets are reported.

Table 5 and Figure 8 showed a good agreement between experimental and predicted SWIR for all parameters. Dry matter showed high coefficients of determination (R^2^ = 0.981 for the training test and R^2^ = 0.924 for the test set) and values of the other metrics close to 0. An optimal prediction performance resulted for titratable acidity (R^2^ = 0.987 for the training test and R^2^ = 0.953 for the test set) and total soluble solid content (R^2^ = 0.965 for the training test and R^2^ = 0.898 for the test set). The model for firmness was promising, with R^2^ being equal to 0.932 for the training test and R^2^ being equal to 0.820 for the test set, and showing low values of MAE. However, a large dispersion of residuals, i.e., high RMSE, indicated a discrete prediction performance of the models (RMSE = 7.191 for the training test and RMSE = 10.774 for the test set). The discrete performance of the FF model for the SWIR system may be due to the reasons indicated for the VisNIR calibration. However, compared to the latter, the SWIR model for firmness was better. This can be explained by the fact that the SWIR spectral region considered the O-H vibrational modes of all NIR spectrum overtones. These modes are related to the fruit structure, and therefore to the firmness.

In order to explain the performances of the models in predicting the pomological traits using the two hyperspectral systems, a sensitivity analysis was carried out. This analysis highlighted the relative importance of the input variables to ANN model predictions. The normalized relative variable importance of the projection (VIP) across the spectral region for each of the ANN model was reported in Table 6 and Table 7.

Regarding the firmness model, the spectra bands, which strongly contributed to the prediction capability, ranged from 450–880 nm (Table 6). As previously reported by Merzlyak and Solovchenko (2002) [47], the VIS region (450–700 nm) is strongly related to the absorption of pigment (such as chlorophylls, carotenoids, anthocyanins, etc.), whose concentration depends on the fruit ripening stage. Moreover, the spectral region around 800–950 nm also contributed to the firmness prediction: this region is an indicator of the probable sugar and water absorption, which varies during fruit softening, as reported by Pu et al. (2016) [39]. Similarly to what was found for FF, the spectral regions that most influenced the predictive capacity of the TSS model were those related to pigments and carbohydrates (C–H third overtone). For example, the absorption bands between 570 and 590 nm are related to carotenoids, and those between 680 and 710 nm to chlorophyll-α [39,48,49]. In fact, many authors highlighted a close relationship between fruit pigment variation, soluble solid content, and ripening stage [48,49,50]. The bands from 800 to 1000 nm contributed to the TSS model. This band is associated with the water absorption peak [36,37]. The bands from 800 to 1000 nm are strongly related to the O–H water overtone and second overtone and the O–H bonds’ combination band and C–H third overtone [41]. For TA model, the Vis region had great importance because fruit acidity is strongly related to the maturation stage [49]. In addition, absorbance contribution at 950 nm is due to the O–H overtone of organic acids. For DM model, the absorbances from 760 to 970 nm were among the most important predictive variables because they are related to the O–H vibration of water. 

For the SWIR system, VIP spectral regions are reported in Table 8. Firmness was strongly related to the spectral regions between 1123 and 1361 nm, which could be ascribed by the O–H second overtone O–H bonds’ combination band, and the C–H second and third overtones. These functional groups are characteristic to the water and carbohydrates, which are strongly associated with the fruit softness. However, other spectral bands near 1200 nm, 1300 nm, 1400 nm, and 1700 nm, characteristic of the C–H stretching second overtone and O–H functional groups [46], were predictive for FF. These spectral regions were the most predictive also for the TSS model, because they are characteristic of sugar functional groups, the main constituent of soluble solid content. The absorption peak near 1344 nm was considered the most important spectral band in the TA model, because it is related to the C–H and O–H functional groups of organic acids. Moreover, the spectral bands near 1300 and 1600 nm, characteristic of O–H overlapping with the first overtones of the O–H stretching and water-bonded O–H functional groups, also contributed to the TA model. As for VisNIR, the bands characteristic of the O–H group of the water at around 1400 nm [46] were the main predictors of the DM model.

Regarding the fused dataset, the best model for firmness was carried out with 13 neurons in the hidden layer, a hyperbolic tangent activation function for the hidden neurons, and a logistic activation function for the output neurons (Table 8). The optimal model for total soluble solid content was obtained with 17 neurons in the hidden layer, an exponential activation function for the hidden neurons, and an identity activation function for the output neurons. The best model of titratable acidity had 15 neurons in the hidden layer, an identity activation function for the hidden neurons, and a logistic activation function for the output neurons. Finally, the best model for dry matter was characterized by 11 neurons in the hidden layer, a logistic activation function for hidden neurons, and an identity activation function for output neurons.

**Figure 9:**
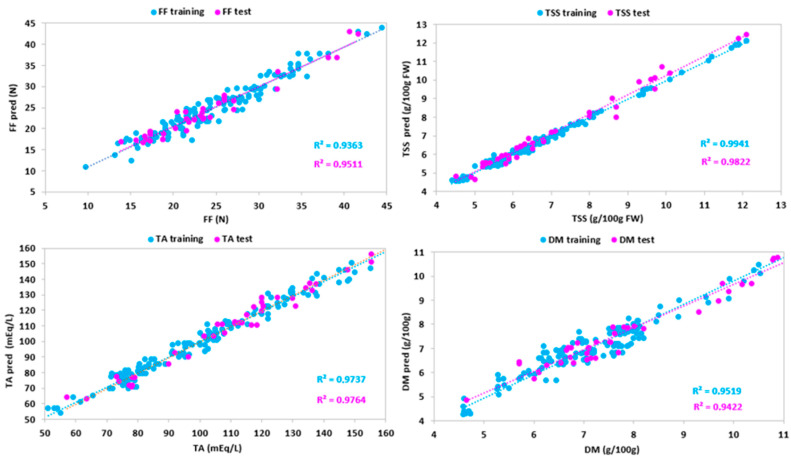
Predicted vs. experimental values of the firmness (FF), total soluble solid content (TSS), titratable acidity (TA), dry matter (DM) using the optimal ANN topologies and spectra from data fusion. The coefficients of determination (R^2^) for training and test sets are reported.

The predictive performances of all models built by the fusion band spectral features were better than that of the single-band spectral features (Table 8 and Figure 9), especially for the firmness, whose model showed higher metrics (R^2^ = 0.951, RMSE = 1.554, MAE = 0.343 and RSE = 6.602 for the test set). This result demonstrated that the information obtained from the two his systems can have a synergistic effect, improving the model’s predictiveness.

Our study showed how the two HSI systems can predict the internal quality of strawberries. However, the predictive capacity may also depend on the quality of the hyperspectral data, as well as on the algorithms used for prediction. In particular, a high imaging speed can reduce spectral and spatial resolution. The optimal calibration of these parameters is strictly linked to the structure of the instrument. To monitor real-time dynamic biological samples with high spectral and spatial resolution, it is possible to use a stand-down video-rate high-throughput HSI, equipped with a high-speed galvo mirror, that allows one to scan spatial light, obtaining a spectral resolution of 3–5 nm and accelerating the collection rate of hyperspectral cubes to the video level, as described by Li et al. [51].

## 4. Conclusions

In this study, the potential of hyperspectral imaging in both VisNIR and SWIR regions for predicting the strawberries’ pomological traits was investigated. We demonstrated that the hyperspectral information obtained using the two systems on different cultivars of strawberries exhibited potential for the prediction of the firmness, total soluble solids content, titratable acidity, and dry matter of strawberries. Comparing the performances of the two systems, our results indicated similar predictive power of models for total soluble solid content, titratable acidity, and dry matter, while the model from SWIR system showed a better performance for firmness. Finally, data fusion improved performances of all models, especially for firmness.

The overall results showed that the HSI technique represents a rapid, nondestructive alternative for the quality assessment of strawberries, which would benefit the fresh market and food industry. Further research may be aimed toward the use of this kind of system for the real-time prediction and classification of the strawberries in a sorting line.

## Figures and Tables

**Table 1:** Cultivars, pedigree, origin, cultivation area (from: https://plantgest.imagelinenetwork.com; accessed on 18 December 2023) for the four strawberry cultivars.

Cultivar	Pedigree	Origin
Sabrina	Sel. 90-020-01 × Sel. 97-19	Spain
Calinda	Unknown	Netherlands and Bonares, Andalusia, Spain
Marimbella	Unknown	Italy
Sabrosa-Candonga	Sel. 92-38 × Sel. 86-032	Spain

**Table 2:** Means and standard deviation of quality parameters of first set (VisNIR) samples divided by cultivar. Differences between letters (a, b, c) in the same row indicate significant differences (*p* < 0.05).

	Sabrina	Calinda	Marimbella	Sabrosa
Weight (g)	57 ± 13 a	48 ± 14 a	46 ± 16 a	22 ± 10 b
Length (mm)	71 ± 10 a	60 ± 5 a	64 ± 10 ab	46 ± 9 b
Width (mm)	48 ± 5 a	46 ± 5 a	45 ± 7 a	34 ± 5 b
Thickness (mm)	34 ± 2 b	40 ± 3 a	37 ± 5 ab	31 ± 4 b
L*	33.17 ± 2.47 a	35.55 ± 3.56 a	35.28 ± 3.69 a	39.56 ± 4.03 a
a*	27.24 ± 4.31 b	31.27 ± 3.55 ab	32.71 ± 3.99 ab	34.54 ± 3.46 a
b*	16.09 ± 5.98 a	16.15 ± 4.59 a	19.93 ± 5.89 a	24.96 ± 5.46 a
FF (N)	25 ± 6 a	24 ± 4 a	28 ± 8 a	21 ± 6 a
TSS (g 100 g^−1^ FW)	5.9 ± 0.7 bc	5.4 ± 0.5 c	6.8 ± 0.7 ab	9 ± 2 a
TA (mEq 100 L^−1^)	89 ± 13 b	76 ± 12 b	113 ± 15 a	128 ± 18 a
DM (g 100 g^−1^ FW)	6.8 ± 0.9 a	7.7 ± 0.4 a	6.5 ± 0.7 a	8.8 ± 2.7 a

Legend: FF = firmness; TSS = total soluble solid content; TA = titratable acidity; DM = dry matter; FW = fresh weight; L*, a*, b* = CIELab coordinates.

**Table 3:** Means and standard deviation of quality parameters of second set (SWIR) samples divided by cultivar. Differences between letters (a, b, c) in the same row indicate significant differences (*p* < 0.05).

	Sabrina	Calinda	Marimbella	Sabrosa
Weight (g)	27 ± 10 b	48 ± 14 ab	58 ± 10 a	35 ± 9 b
Length (mm)	54 ± 6 b	59 ± 5 b	72 ± 7 a	58 ± 8 ab
Width (mm)	35 ± 6 b	46 ± 5 a	50 ± 4 a	40 ± 4 b
Thickness (mm)	31 ± 4 b	40 ± 3 a	37 ± 3 ab	35 ± 4 ab
L*	34.81 ± 4.11 a	35.47 ± 3.51 a	36.32 ± 3.80 a	36.09 ± 4.95 a
a*	30.56 ± 4.33 a	33.28 ± 3.52 a	34.24 ± 2.45 a	33.93 ± 2.79 a
b*	18.48 ± 6.68 a	16.57 ± 4.68 a	18.20 ± 5.37 a	25.26 ± 7.14 a
FF (N)	28 ± 9 a	24 ± 5 a	27 ± 7 a	24 ± 5 a
TSS (g 100 g^−1^ FW)	6.4 ± 0.5 a	5.4 ± 0.6 a	6.6 ± 0.8 a	6.8 ± 1.2 a
TA (mEq L^−1^)	112 ± 11 a	75 ± 9 b	103 ± 20 a	111 ± 16 a
DM (g 100 g^−1^ FW)	6.2 ± 0.8 b	7.7 ± 0.3 a	6.7 ± 0.7 b	6.5 ± 1.2 b

Legend: FF = firmness; TSS = total soluble solid content; TA = titratable acidity; DM = dry matter; FW = fresh weight; L*, a*, b* = CIELab coordinates.

**Table 4:** Visible–near-infrared spectrometer. Neural network architectures, regression metrics for the highest training and test set predictions, goodness of fit, and residual analysis for the developed ANN models.

	Neural Network Topologies (Input–Hidden–Output)	Activation Function		Training Set				Test Set			
		Hidden Neurons	Output Neurons	R^2^	RMSE	MAE	RSE	R^2^	RMSE	MAE	RSE
FF	MLP (204–11–1)	Logistic	Identity	0.682	3.577	0.014	14.443	0.808	3.493	0.484	14.234
TSS	MLP (204–22–1)	Exp	Tanh	0.967	0.317	0.002	4.720	0.959	0.394	0.067	5.544
TA	MLP (204–16–1)	Identity	Identity	0.973	3.922	0.019	3.959	0.877	9.306	1.003	8.438
DM	MLP (204–19–1)	Exp	Exp	0.967	0.315	0.013	4.273	0.947	0.380	0.038	4.911

Legend: MLP = multilayer perceptron; Tanh = hyperbolic tangent function; Exp = exponential function; FF = firmness (N); TSS = total soluble solid content (g 100 g^−1^ FW); TA = titratable acidity (mEq L^−1^); DM = dry matter (g 100 g^−1^ FW).

**Table 5:** Short-wave infrared spectrometer. Neural network architectures, regression metrics for the highest training and test set predictions, goodness of fit, and residual analysis for the developed ANN models.

	Neural Network Topologies (Input–Hidden–Output)	Activation Function		Training Set				Test Set			
		Hidden Neurons	Output Neurons	R^2^	RMSE	MAE	RSE	R^2^	RMSE	MAE	RSE
FF	MLP (224–10–1)	Logistic	Identity	0.932	1.896	0.041	7.191	0.820	2.740	0.313	10.774
TSS	MLP (224–11–1)	Logistic	Identity	0.965	0.187	0.008	2.995	0.898	0.297	0.029	4.593
TA	MLP (224–19–1)	Logistic	Exp	0.987	2.396	0.157	2.387	0.953	4.838	1.069	4.869
DM	MLP (224–11–1)	Logistic	Exp	0.981	0.135	0.003	1.984	0.924	0.306	0.018	4.542

Legend: MLP = multilayer perceptron; Tanh = hyperbolic tangent function; Exp = exponential function; FF = firmness (N); TSS = total soluble solid content (g 100 g^−1^ FW); TA = titratable acidity (mEq L^−1^); DM = dry matter (g 100 g^−1^ FW).

**Table 6:** Normalized relative importance (%) of the most significative input variables (wavelengths λ) to ANN model predictions for VisNIR system.

FF		TSS		TA		DM	
λ (nm)	%	λ (nm)	%	λ (nm)	%	λ (nm)	%
951	100.0	820	100.0	951	100.0	914	100.0
516	98.1	664	99.8	441	98.4	939	99.8
706	97.9	878	99.6	566	98.1	799	99.4
637	97.7	616	99.1	505	98.0	679	99.4
905	97.7	781	97.9	799	97.9	551	99.3
826	9.6	449	97.1	670	97.6	432	98.9
581	97.3	619	97.0	679	97.4	655	98.8
670	97.2	513	96.7	513	97.4	528	97.9
569	97.2	1000	96.6	930	97.3	455	97.7
784	96.8	887	96.3	418	97.3	634	97.7

Legend: FF = firmness; TSS = total soluble solid content; TA = titratable acidity; DM = dry matter.

**Table 7:** Normalized relative importance (%) of the most significative input variables (wavelengths λ) to ANN model predictions for SWIR system.

FF		TSS		TA		DM	
λ (nm)	%	λ (nm)	%	λ (nm)	%	λ (nm)	%
1123	100.0	1379	100.0	1344	100.0	1588	100.0
1120	98.4	1134	100.0	1588	99.6	1263	99.9
1361	98.0	1567	99.9	1210	97.9	1404	99.2
1649	97.7	1291	99.7	1365	97.3	981	99.1
1071	97.7	1288	98.2	1186	97.3	974	98.9
1270	97.3	1464	97.9	1535	97.3	977	98.8
991	96.9	1404	97.8	1081	97.0	938	98.5
1249	96.7	1295	97.6	998	96.9	1471	98.5
1602	96.6	1556	97.4	1266	96.5	1379	98.2
1383	96.6	1228	97.4	1193	96.3	1088	98.2

Legend: FF = firmness; TSS = total soluble solid content; TA = titratable acidity; DM = dry matter.

**Table 8:** Visible–near-infrared and Short-wave infrared and spectrometers. Neural network architectures, regression metrics for the highest training and test set predictions, goodness of fit, and residual analysis for the developed ANN models.

	Neural Network Topologies (Input–Hidden–Output)	Activation Function		Training Set				Test Set			
		Hidden Neurons	Output Neurons	R^2^	RMSE	MAE	RSE	R^2^	RMSE	MAE	RSE
FF	MLP (406–13–1)	Tanh	Logistic	0.936	1.609	0.180	6.495	0.951	1.554	0.343	6.602
TSS	MLP (406–17–1)	Exp	Identity	0.994	0.134	0.003	1.996	0.981	0.309	0.132	4.353
TA	MLP (406–15–1)	Identity	Logistic	0.974	3.896	0.027	3.933	0.976	4.018	0.426	3.643
DM	MLP (406–11–1)	Logistic	Identity	0.952	0.399	0.125	5.420	0.942	0.415	0.108	5.361

Legend: MLP = multilayer perceptron; Tanh = hyperbolic tangent function; Exp = exponential function; FF = firmness (N); TSS = total soluble solid content (g 100 g^−1^ FW); TA = titratable acidity (mEq L^−1^); DM = dry matter (g 100 g^−1^ FW).

## Data Availability

Since the data were not included in an open access repository, the results may not be reproducible by other researchers.

## Supplementary Materials

The following supporting information can be downloaded at: https://www.mdpi.com/article/10.3390/s24010174/s1, Table S1: Visible Near Infrared spectrometer. Neural network architectures and correlation coefficients for the developed ANN models; Table S2: Short Wave Infrared spectrometer. Neural network architectures and correlation coefficients for the developed ANN models.
