# Supplementary material for: A Performance Evaluation of Two Hyperspectral Imaging Systems for the Prediction of Strawberries’ Pomological Traits
Source: Sensors (Basel). 2023 Dec 28;24(1):174. doi: 10.3390/s24010174 (PMC10781302; doi:10.3390/s24010174)
Supplement: Supplementary file 1 [file sensors-24-00174-s001.zip › sensors-2747508-supplementary.pdf]

Supplementary Table S1. Visible Near Infrared spectrometer. Neural network architectures and correlation coefficients for the developed ANN models. The best architecture for each parameter was highlighted in bold.

	Neural Network Topologies (input-hidden-output)	Activation Function		Training Set	Test Set
		Hidden Neurons	Output Neurons	Correlation coefficient	Correlation coefficient
FF	**MLP (204–11–1)**	**Logistic**	**Identity**	**0.8258**	**0.8989**
	MLP (204–21–1)	Logistic	Logistic	0.6944	0.6546
	MLP (204–14–1)	Tanh	Logistic	0.6149	0.5642
	MLP (204–11–1)	Exp	Exp	0.7011	0.6620
	MLP (204–16–1)	Tanh	Exp	0.5951	0.5525z
TSS	**MLP (204–22–1)**	**Exp**	**Tanh**	**0.9834**	**0.9793**
	MLP (204–9–1)	Tanh	Logistic	0.9653	0.9278
	MLP (204–8–1)	Tanh	Tanh	0.9752	0.9209
	MLP (204–19–1)	Tanh	Identity	0.9814	0.9230
	MLP (204–11–1)	Exp	Tanh	0.9884	0.9193
TA	**MLP (204–16–1)**	**Identity**	**Identity**	**0.9864**	**0.9365**
	MLP (204–9–1)	Identity	Exp	0.9710	0.9216
	MLP (204–9–1)	Identity	Logistic	0.9832	0.9273
	MLP (204–19–1)	Identity	Identity	0.9867	0.9358
	MLP (204–22–1)	Identity	Exp	0.9707	0.9221
DM	**MLP (204–19–1)**	**Exp**	**Exp**	**0.9834**	**0.9731**
	MLP (204–21–1)	Tanh	Exp	0.8869	0.8743
	MLP (204–22–1)	Tanh	Exp	0.9059	0.9052
	MLP (204–23–1)	Logistic	Exp	0.9094	0.9188
	MLP (204–20–1)	Exp	Logistic	0.8595	0.8941

Legend: MLP = multilayer perceptron; Tanh = hyperbolic tangent function; Exp = exponential function; FF = firmness (N); TSS = total soluble solid content (g 100 g^{-1} FW); TA = titratable acidity (mEq L^{-1}); DM = dry matter (g 100 g^{-1} FW).

Supplementary Table S2. Short Wave Infrared spectrometer. Neural network architectures and correlation coefficients for the developed ANN models. The best architecture for each parameter was highlighted in bold.

	Neural Network Topologies (input-hidden-output)	Activation Function		Training Set	Test Set
		Hidden Neurons	Output Neurons	Correlation coefficient	Correlation coefficient
FF	**MLP (224–10–1)**	**Logistic**	**Identity**	**0.9654**	**0.9055**
	MLP (224–25–1)	Tanh	Identity	0.9611	0.8282
	MLP (224–23–1)	Identity	Logistic	0.9711	0.8995
	MLP (224–9–1)	Identity	Identity	0.9780	0.8162
	MLP (224–10–1)	Tanh	Logistic	0.9214	0.8870
TSS	**MLP (224–11–1)**	**Logistic**	**Identity**	**0.9823**	**0.9476**
	MLP (224–14–1)	Identity	Tanh	0.9754	0.9401
	MLP (224–14–1)	Identity	Identity	0.9768	0.9458
	MLP (224–10–1)	Logistic	Tanh	0.9699	0.9400
	MLP (224–13–1)	Logistic	Identity	0.9768	0.9422
TA	**MLP (224–19–1)**	**Logistic**	**Exp**	**0.9935**	**0.9762**
	MLP (224–18–1)	Logistic	Exp	0.9605	0.9041
	MLP (224–22–1)	Tanh	Identity	0.9484	0.9036
	MLP (224–13–1)	Tanh	Logistic	0.9719	0.9022
	MLP (224–20–1)	Logistic	Identity	0.9581	0.9272
DM	**MLP (224–11–1)**	**Logistic**	**Exp**	**0.9905**	**0.9612**
	MLP (224–25–1)	Logistic	Tanh	0.9954	0.9323
	MLP (224–21–1)	Logistic	Tanh	0.9682	0.9585
	MLP (224–19–1)	Tanh	Identity	0.9955	0.9440
	MLP (224–16–1)	Tanh	Tanh	0.9702	0.9584

Legend: MLP = multilayer perceptron; Tanh = hyperbolic tangent function; Exp = exponential function; FF = firmness (N); TSS = total soluble solid content (g 100 g^{-1} FW); TA = titratable acidity (mEq L^{-1}); DM = dry matter (g 100 g^{-1} FW).
